# The precipitate structure of copper-based antibacterial and antiviral agents enhances their longevity for kitchen use

**DOI:** 10.1038/s41538-024-00324-4

**Published:** 2024-10-25

**Authors:** Takashi Nishimura, Masami Hashimoto, Kageto Yamada, Ryuji Iwata, Kazuhiro Tateda

**Affiliations:** 1 Saitama Industrial Promotion Public Corporation, Shintoshin Business Exchange Plaza 3F, 2-3-2 Kamiochiai, Chuo-ku, Saitama City, Saitama Prefecture 338-0001 Japan; 2 Materials Research and Development Laboratory, Japan Fine Ceramics Center, 2-4-1 Mutsuno, Atsuta-ku, Nagoya, 456-8587 Japan; 3 Department of Microbiology and Infection Diseases, Toho University, 5-21-16 Omorinishi, Ota-ku, Tokyo, 1143-8540 Japan; 4 Department of Technology Management for Innovation, School of Engineering, The University of Tokyo, 7-3-1 Hongo, Bunkyo-ku, Tokyo, 113-0033 Japan

**Keywords:** Structural properties, Antimicrobial responses

## Abstract

The transmission of bacteria through cooking surfaces, the handles of hot plates, and cookware that is not cleaned frequently can pose a problem. In this study, a copper ion-based mixed solution (CBMS) containing only inorganic ions with controlled acidity was assessed as a new antibacterial and antiviral agent. We analysed the structure of the precipitates, and various deposits measuring a few micrometres were observed on the substrates. We have defined these deposits as strongly bonded scaly copper dispersion (SBSCD) structures.

The antibacterial copper component of the liquid agent changed over time after application; this mechanism appears to be responsible for the maintenance of antibacterial performance.

CBMS demonstrates high safety for the human body and can be applied to stainless steel materials used in kitchens and tables. It exhibits a sustained antibacterial effect over time, and its antibacterial properties can be continuously maintained.

## Introduction

The global COVID-19 pandemic has heightened public awareness of infectious diseases, which have become a significant social concern. Governments worldwide are urging the development of preventive measures to curb infections within the food environment^1^. In particular, the food service industry has implemented various sterilization and virus elimination methods, employing antibacterial and antiviral agents such as ethanol-based disinfectants, sodium hypochlorite, photocatalysts, and silver or copper ions^2^. For example, in Japan, hypochlorous acid water, produced through the electrolysis of hydrochloric acid or NaCl water, has gained approval as a food additive. It serves as an affordable, safe, and reliable disinfectant for fresh food^3,4^.

On the other hand, bacterial transmission uncleaned cooking surfaces such as tables, hot plate handles, and cookware poses a significant concern^5–7^. Commercial countertops and utensils, often crafted from SUS304 (UNS S30400), a stainless steel material resistant to rust, can harbour bacteria if not sanitized regularly.

Additionally, hospital-acquired infections, particularly those caused by drug-resistant bacteria such as methicillin-resistant *Staphylococcus aureus* (MRSA), remain a prevalent issue in healthcare settings^8,9^. Rapid and long-lasting sterilization of various environmental surfaces in hospitals is necessary to combat such infections.

Furthermore, copperware and silverware have served as containers for storing water since ancient times^10,11^.

Copper and silver exhibit antibacterial properties that have been extensively documented in various publications^12–17^.

The antibacterial effects of copper ions are attributed to several mechanisms, including damage to cell membranes^18,19^, the generation of active oxygen^20–24^, DNA damage^25,26^, and photocatalytic effects^27–30^.

Studies have also been published on the antiviral properties of copper and copper oxide^31^. Furthermore, there have been specific investigations into the effectiveness of these materials against SARS-CoV-2^32,33^.

SKYBE-783^TM^, a novel antibacterial and antiviral agent developed in Japan, is a transparent liquid formulation that can be easily sprayed onto the surfaces of diverse shapes and materials. This liquid agent, known as a copper ion-based mixed solution (CBMS), comprises only inorganic ions, including Cu^2+^ (140 mg/l), Ca^2+^ (0.11 mg/l), Cl^−^ (33 mg/l), and HPO_4_^2−^ (12.0 mg/l), and has a pH value close to 3.0.

Antibacterial and antiviral effects are exerted relatively quickly; in particular, antiviral effects have been confirmed to appear within 30 min and last for a long time. This enduring effect is attributed to the combined action of various copper-based compounds, which precipitate upon drying of the liquid agent on the surface.

Our analysis focused on examining the structure and composition of the precipitates formed, aiming to elucidate the mechanism responsible for the antibacterial and antiviral activities exhibited by this liquid agent.

To investigate the various antibacterial and antiviral properties of the precipitates, *Escherichia coli* and *Staphylococcus aureus* were selected as representative bacteria known to cause food poisoning.

The liquid agent was applied by spraying onto polymethyl methacrylate (PMMA), SUS304 (UNS S30400), and SUS316L (UNS S31603) substrates commonly utilized in cooking and food service environments. We then evaluated the antibacterial properties of the treated substrates at intervals of 8 days, 14 days, 3 months, 6 months, and 1 year after application. This allowed us to study the changes in antibacterial properties over time.

To assess the effectiveness against MRSA in medical settings, we chose stainless steel SUS304, acrylic resin, and a PC keyboard as representative materials commonly encountered in such environments.

After the liquid agent was applied, these materials were maintained at room temperature and humidity levels for durations ranging from 1 to 12 weeks. Subsequently, they were exposed to MRSA for a period of 2 h, and the sterilization performance was evaluated.

Additionally, we present the results of an adenosine triphosphate (ATP) test, which serves as an alternative to traditional antibacterial tests. This test was conducted both before and after application of the liquid agent in a kitchen setting.

## Results

### Structure and composition analysis of the precipitates

Liquid agent-coated surfaces were prepared by spraying 20 ml/m^2^ onto various substrates (PMMA, SUS304, SUS316L, and Zr) and then left to dry for different durations ranging from 8 days to 1 year at room temperature.

**Fig. 1:**
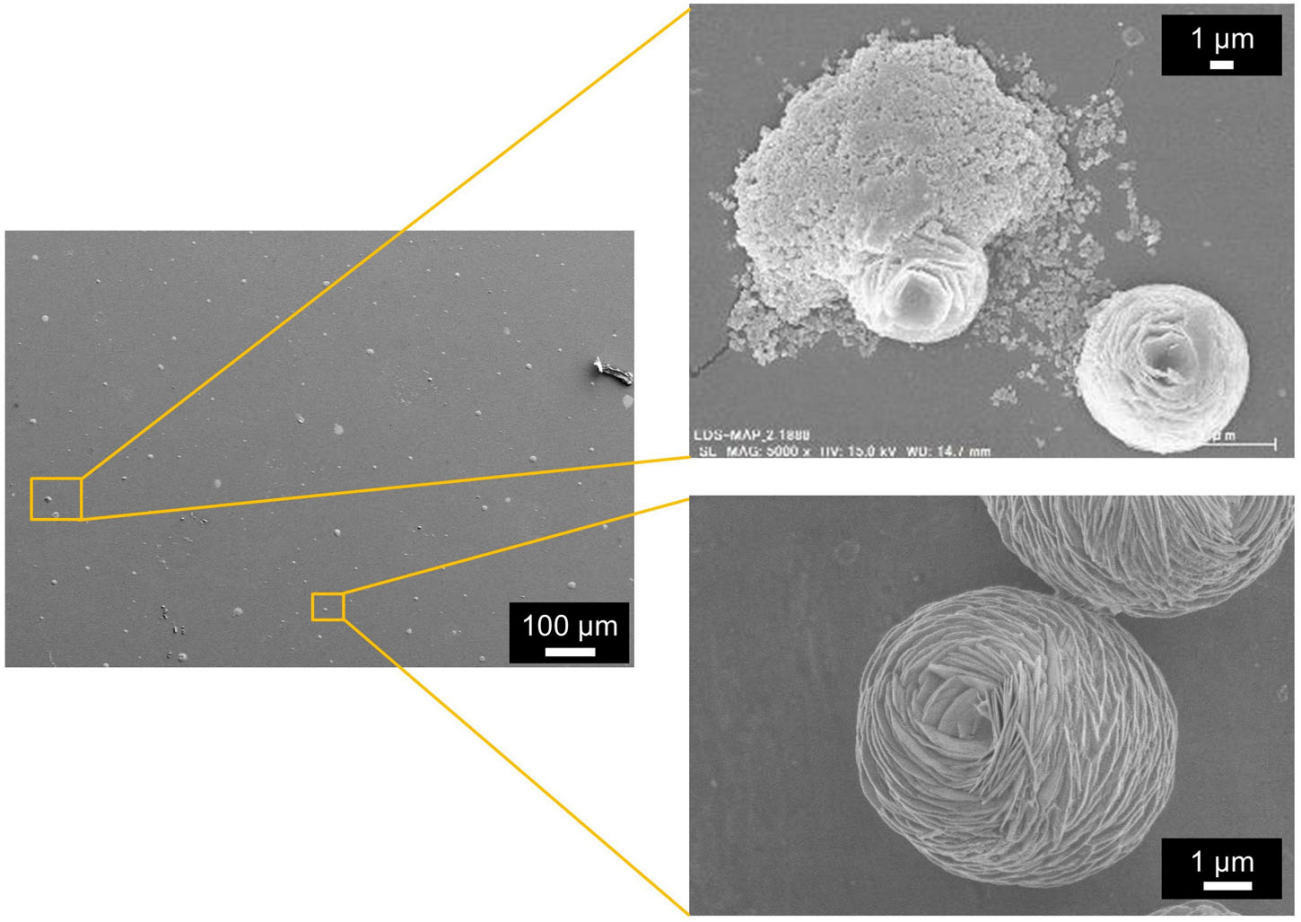
SEM images of the precipitates of the liquid agent (copper ion-based mixed solution, CBMS) on the PMMA substrate after drying for 20 days at room temperature.

Figure 1 shows scanning electron microscopy (SEM) images of the precipitates of CBMS on the PMMA substrate after drying for 20 days at room temperature.

Deposits with a size of approximately 5 μm were observed on the substrate, exhibiting a varied microstructure. For example, we observed agglomerates consisting of submicron particles resembling scaly skin, as well as flower-shaped precipitates measuring approximately 5 μm in size.

**Fig. 2:**
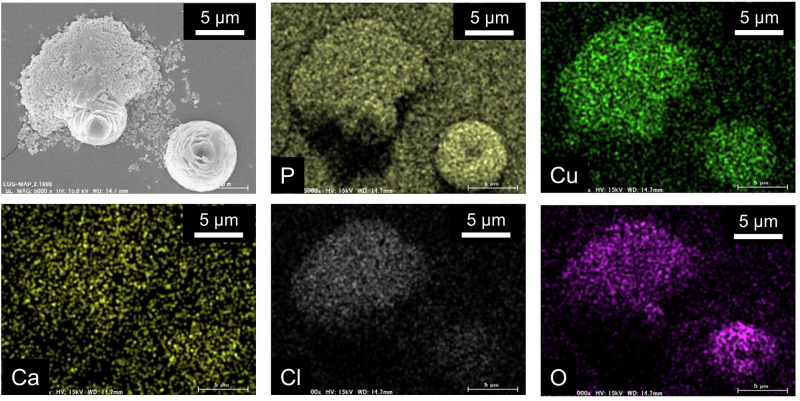
SEM-EDX images of each constituent element of the precipitate of CBMS shown in the upper right of Fig. 1.

Figure 2 presents scanning electron microscope-energy-dispersive X-ray spectroscopy (SEM-EDX) images of the precipitates depicted in Fig. 1, with the aim of identifying the constituent elements of the precipitates with different shapes. This SEM‒EDX analysis provides valuable insights into the chemical composition and mixing state of the materials. The agglomerates composed of submicron particles contained constituents such as Cu, Cl, and O, whereas the flower-shaped precipitates contained constituents such as Cu, P, and O. Interestingly, the flower-shaped precipitates resemble previously published SEM images of Cu_2_(OH)PO_4_ and CuO^27,34^.

However, due to the 1 μm spatial resolution of SEM-EDX, it is challenging to discern correlations between different elements. To address this limitation, scanning transmission electron microscopy–energy-dispersive X-ray spectroscopy (STEM–EDS) with a spatial resolution of 5 nm was employed.

For cross-sectional observation, substrates coated with CBMS were embedded in resin and thinned using a focused ion beam scanning electron microscope (FIB-SEM) system, followed by sample preparation for transmission electron microscopy (TEM) analysis.

**Fig. 3:**
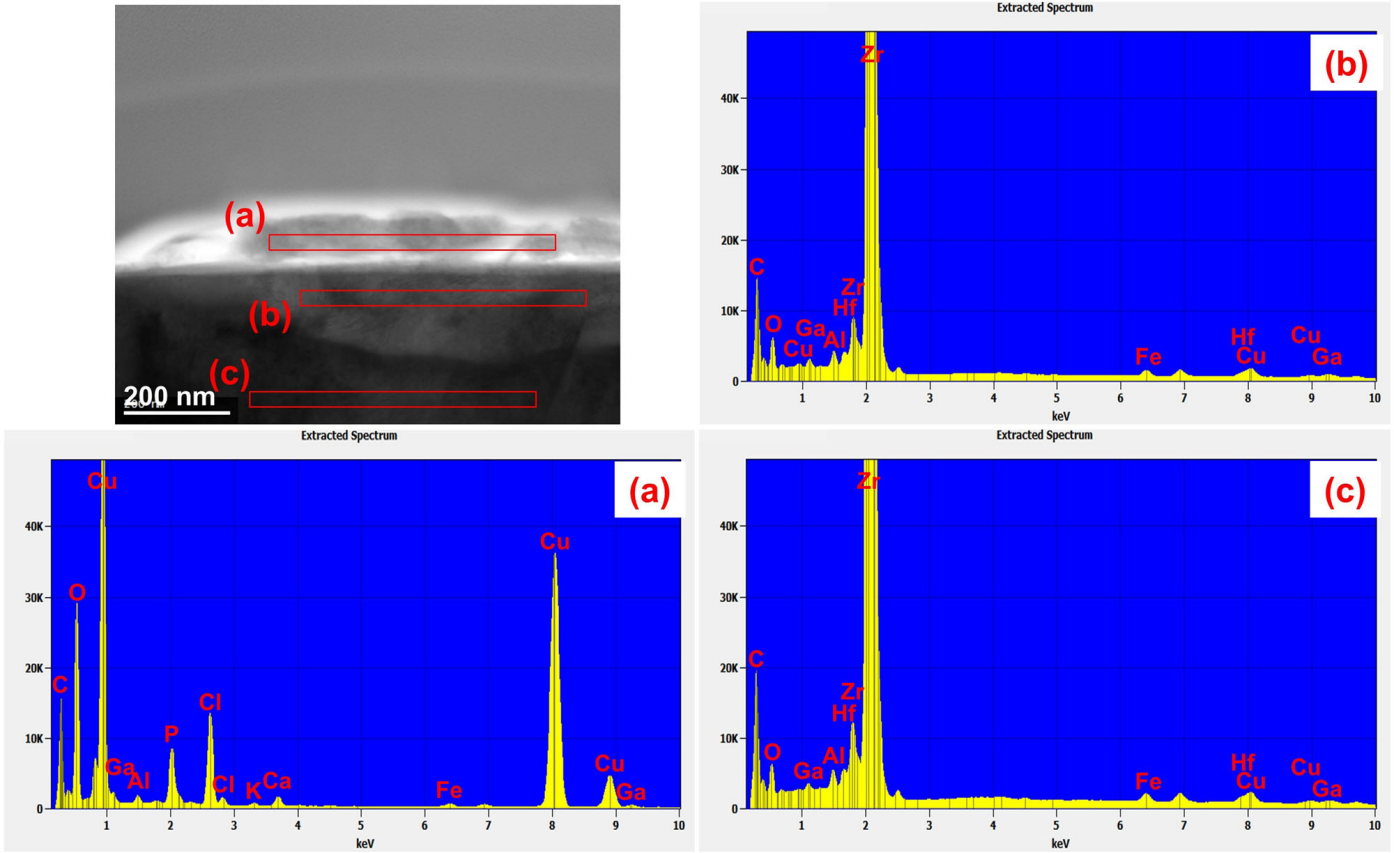
STEM image of the cross-section of precipitate on Zr dried for 20 days after CBMS application at room temperature. EDS results of **a** precipitate on the Zr substrate, **b** the substrate at a depth just below the surface, and **c** the Zr substrate.

Figure 3 shows the results of STEM-EDS analysis of (a) the precipitate on the Zr substrate after drying for 20 days at room temperature, (b) the substrate at a depth just below the surface, and (c) the Zr substrate itself. Elements such as Cu, P, Cl, and O, which are not found inside the Zr substrate, were detected in the precipitates. On the basis of the findings in Fig. 2 and Fig. 3, it can be inferred that the precipitates on both the PMMA and Zr substrates had the same composition of Cu, P, Cl, and O.

**Fig. 4:**
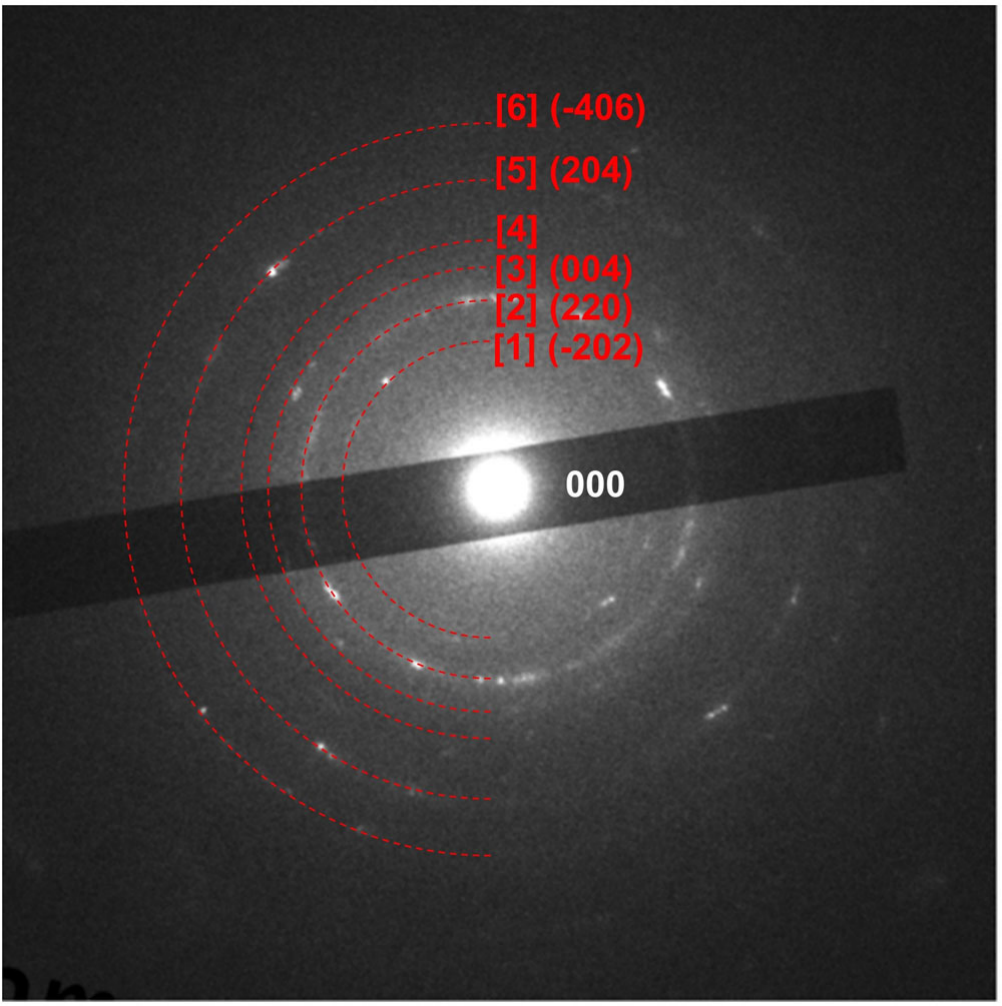
Electron diffraction patterns of (a) the precipitate on Zr dried for 20 days after CBMS application as shown in Fig. 3. P_2_ The face index of Cu_2_P_2_O_7_ is shown in [1], [2], [3], [5] and [6]. [4] is the face index of Cl_2_Cu_2_O.

The small amount of precipitated product on the substrates after storage at room temperature precluded measurement via X-ray diffraction. Figure 4 shows the electron diffraction pattern of the precipitate (a) depicted in Fig. 3. The crystal structure of the precipitate was assigned to various copper compounds, including Cu_2_P_2_O_7_, Cu_2_O and Cl_2_Cu_2_O. These copper compounds were inferred to have formed via the dissolution and precipitation of ions present in CBMS. The diffraction patterns are not clear due to the small number of crystallites in the precipitates and their low crystallinity. Many of the diffraction patterns were consistent with those of Cu_2_P_2_O_7_: the (-202) plane in [1], the (220) plane in [2], the (004) plane in [3], the (204) plane in [5], and the (-406) plane in [6]. The diffraction patterns of [2] and [3] were also consistent with the diffraction patterns of Cu_2_O and Cl_2_Cu_2_O; [2] was in the (211) plane of Cu_2_O and (222) plane of Cl_2_Cu_2_O, and [3] was in the (220) plane of Cu_2_O and (331) plane of Cl_2_Cu_2_O. Furthermore, [4] coincided with the (133) plane of Cl_2_Cu_2_O, and [6] coincided with the (332) plane of Cu_2_O.

Three crystals matched the obtained electron diffraction patterns, suggesting that the precipitates were a mixture of Cu_2_P_2_O_7_, Cu_2_O and Cl_2_Cu_2_O.

Therefore, the chemical composition of the outermost layer of the precipitated product was analysed using X-ray photoelectron spectroscopy (XPS) with a microprobe. Previous studies have investigated Cu compounds via XPS^35,36^.

**Fig. 5:**
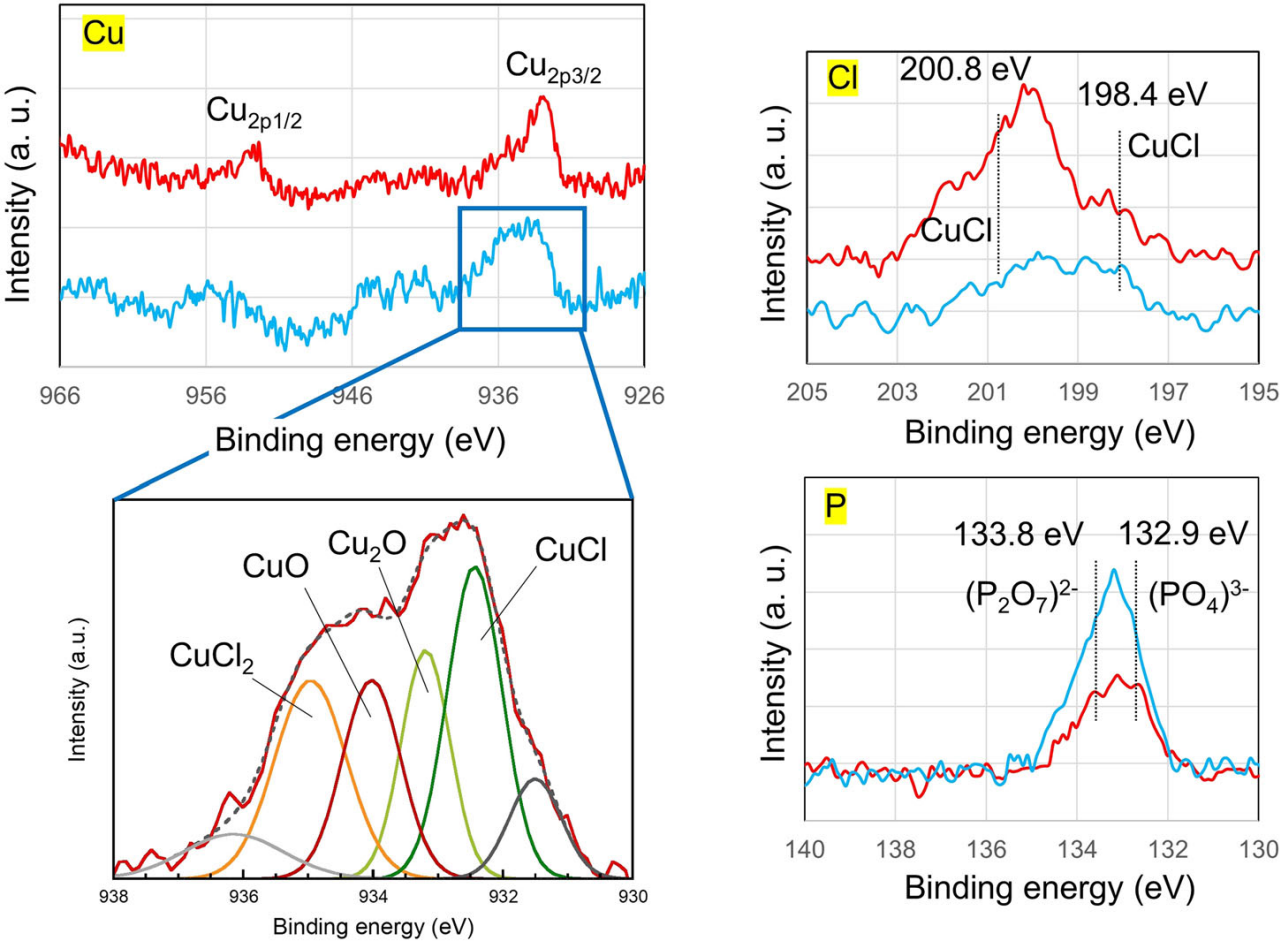
Cu_2p_, P_2p_ and Cl_2p_ XPS spectra of the precipitated products on the SUS304 substrate after drying for 8 days (light blue line) and 1 year (red line) at room temperature.

Figure 5 shows the Cu_2p_, P_2p_ and Cl_2p_ spectra of the precipitated product on the SUS304 substrate after drying for 8 days and 1 year at room temperature. Both the 8-day and 1-year samples presented spectra of copper, chlorine and phosphorus. In the Cu_2p3/2_ level spectrum of the 8-day sample, the binding energies of CuCl, CuCl_2_, Cu_2_O and CuO were 933.2, 935.1, 932.8 and 933.6 eV, respectively^37^. The fractions of CuCl, CuCl_2_, Cu_2_O and CuO determined from the deconvoluted spectra were 26.9, 18.2, 17.4 and 22.4 area %, respectively. The state of Cl in the precipitated product was characterized by using XPS, and the major Cu‒Cl bonds in CuCl were located at 198.4 and 200.8 eV, as shown in Fig. 5 (Cl spectra). Additionally, the state of P in the precipitated product was characterized by using XPS; the peaks of (PO_4_)^3-^ and (P_2_O_7_)^2-^ were located at binding energies of 132.9 and 133.8 eV, respectively. Therefore, chlorine and phosphorus were incorporated into CuO during the drying process, which is consistent with the EDS results shown in Fig. 4. The constituents of the 1-year sample were consistent with those of the 8-day sample. However, the fractions of Cu compounds in the 1-year sample changed. In the 1-year samples, relative to the values for the 8-day samples, the amount of Cl tended to increase, and the amount of P tended to decrease.

Table 1 shows the atom % values of O_1s_, P_2p_, Cl _2p_ and Cu_2p_ in the precipitates with drying time for SUS304 and SUS316L. XPS measurements of the precipitates on PMMA were difficult to obtain due to charge buildup. XPS was used to evaluate a region 0.8 mm in diameter on the substrate. Within this region, the precipitates were scattered within a diameter of 5 μm. Therefore, the area outside the precipitates consisted of the substrate and a large amount of oxygen. For all substrates, with prolonged drying time, the amount of P_2p_ decreased, and the amount of Cl_2p_ increased. In contrast, the Cu content increased until 6 months of drying and then decreased. On the CBMS-coated surface, Cu_2_P_2_O_7_, Cu_2_O and Cl_2_Cu_2_O precipitated together by a dissolution deposition mechanism. At the beginning of deposition, the crystallinity of each crystal was low, and the crystals grew as they dried. Crystallization continued up to a drying time of 6 months, after which the Cu_2p_ content decreased as the larger planes grew.

**Table 1:** Atomic percentages of O_1s_, P_2p_, Cl_2p_ and Cu_2p_ in the precipitates with drying time for SUS304 and SUS316L

	Element (atom %)							
	SUS304				SUS316L			
DryingTime	O_1s_	P_2p_	Cl_2p_	Cu_2p_	O_1s_	P_2p_	Cl_2p_	Cu_2p_
8 days	90.1	7.1	1.7	1.1	95.2	3.4	0.2	1.2
3 months	92.4	5.2	0.2	2.2	93.2	3.3	2.1	2.1
6 months	92.4	1.4	1.3	4.9	92.9	0.8	1.4	3.0
1 year	95.8	1.7	2.2	0.3	93.2	2.3	2.6	1.9

**Fig. 6:**
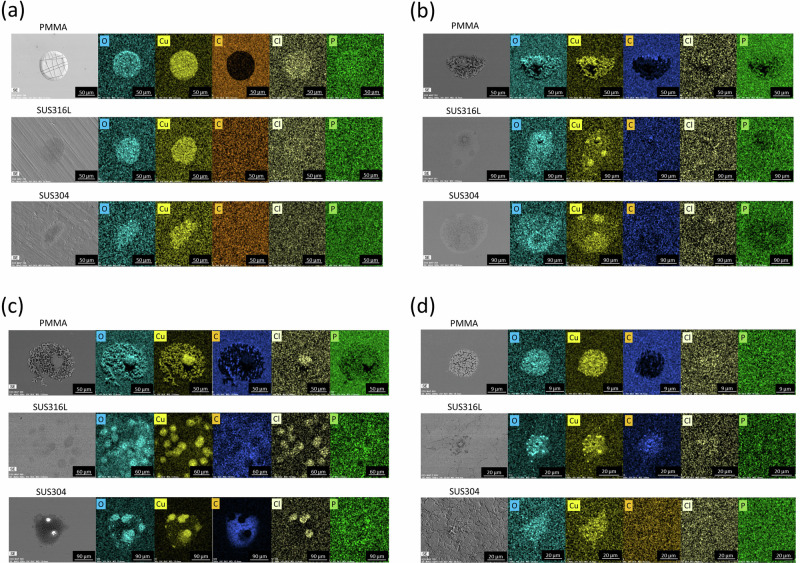
SEM-EDX images of deposits on PMMA, SUS316L and SUS304 according to drying time after CBMS treatment. Deposits of several tens of microns were observed on all substrates. O, Cu, Cl and P components were detected in the precipitates formed on PMMA, SUS316L and SUS304 substrates at each drying time. **a** 8 days, **b** 2 months, **c** 3 months and **d** 1 year.

Because XPS analysis of PMMA was difficult, the trends in SBSCD over time on various substrates were also examined via SEM-EDX. Figure 6 displays SEM‒EDS images of SBSCD on the PMMA, SUS316L, and SUS304 substrates after drying for 8 days to 1 year. Components such as Cu, Cl, P, and O could be observed on the PMMA substrate as well as on SUS304 and SUS316L after drying for 8 days. Even with longer drying times, no differences in the composition of the precipitates were observed between the different substrate types. Therefore, the dependence of antibacterial properties on the substrate type is thought to be related to the number and crystallinity of crystallites and not to the type of precipitate. Cu ions are involved in antibacterial properties, and the elution behaviour of Cu ions is thought to differ depending on crystallinity.

Hydroxyl radicals are likely generated by the Fenton reaction caused by the leaching of Cu ions from SBSCD^23,24^. Consequently, copper compounds deposited on the substrate through liquid agent coating are expected to exhibit effective antibacterial and antiviral activity.

### Various antibacterial and antiviral properties

The following tests were conducted to evaluate the antibacterial and antiviral properties of the CBMS-coated substrates. To assess the antibacterial properties, we employed the antibacterial test method for textile products, considering potential applications such as restaurant seats. Various bacterial strains were used, including *Escherichia coli* NBRC3301, *Escherichia coli* O157　RIMD 0509952, *Staphylococcus aureus* NBRC12732, methicillin-resistant *Staphylococcus aureus* IID1677, *Pseudomonas aeruginosa* NBRC3080, *Moraxella osloensis* ATCC19976, *Klebsiella pneumoniae* NBRC13277, and *Salmonella enterica subsp. Enterica* NBRC3313. The incubation time for all bacteria was 18 h.

Antiviral tests were conducted to measure the antiviral activity on plastics and other nonporous surfaces. Several viruses were tested, including influenza A H3N2 (A/Hong Kong/8/68; TC adapted ATCC VR-1679), Feline calicivirus (FCV) (strain F-9 ATCC VR-782), SARS-CoV-2 (NIID isolate JPN/TY/WK-521), the SARS-CoV-2 alpha mutant strain (hCoV-19/Japan/QK002/2020), the SARS-CoV-2 delta mutant strain (hCoV-19/Japan/TY11-927-P1/2021), and the SARS-CoV-2 omicron mutant strain (hCoV-19/Japan/TY38-873/2021). The culture time for influenza virus and feline calicivirus was 15 min, whereas that for the other viruses was 30 min.

Table 2 shows the results of antibacterial tests on textile products treated with CBMS. The latest test method is ISO20743:2021, but since these tests were conducted in 2020, they followed ISO20743:2013.

**Table 2:** Antibacterial activity value of cotton cloth spray-treated with CBMS

Bacteria	Antibacterial activity value (ISO20743:2013 applicable)	Below Detection limit
*Escherichia coli* NBRC3301	6.2	Yes
*Escherichia coli* O-157 RIMD 0509952	4.7	No
*Staphylococcus aureus* NBRC12732	5.9	No
Methicillin Resistant *Staphylococcus aureus* IID1677	5.8	Yes
*Pseudomonas aeruginosa* NBRC3080	6.0	Yes
*Moraxella osloensis* ATCC19976	6.3	Yes
*Klebsiella pneumoniae* NBRC13277	6.1	Yes
*Salmonella enterica subsp. Enterica* NBRC3313	3.3	Yes

All bacteria except *Staphylococcus aureus*, O-157 and *Salmonella enterica subsp. Enterica* were below the detection limits.

Table 3 shows the results of antiviral tests on precipitated CBMS. Despite the short culture time of 15 to 30 min, the antiviral efficacy values were greater than 3. Hence, it was confirmed that CBMS exhibits excellent antimicrobial and antiviral properties against various bacteria and viruses in a short period.

**Table 3:** Antiviral activity value of substrates treated with CBMS

Virus	Anitviral activity value (ISO21702 applicable)	Contact time
InfluenzaA /H3N2 A/Hong Kong/8/68: TC adapted ATCC VR-1679	5.0	15 min
Feline calicivirus: Strain F-9 ATCC VR-782	3.1	15 min
SARS-Cov-2 NIID isolate JPN/TY/WK-52	3.1	30 min
SARS-Cov-2 Mutant strain (alpha strain) hCoV-19 /Japan/QK002/2020	3.0	30 min
SARS-Cov-2 Mutant strain (delta strain) hCoV-19/Japan/TY11-927-P1/2021	4.3	30 min
SARS-Cov-2 Mutant strain (omicron strain) hCoV-19 /Japan/TY38-873/2021	4.9	60 min

### Sustainability of the antibacterial effect of the liquid agent on *Escherichia coli* and *Staphylococcus aureus* on PMMA, SUS304, and SUS316L

CBMS was applied at 20 ml/m^2^ to the PMMA, SUS304, and SUS316L substrates. After application, the samples were stored at room temperature and humidity for 8 days, 14 days, 3 months, 6 months, or 1 year. Subsequently, antibacterial tests were conducted using the film adhesion method following the JIS Z2801 (ISO 22196:2007) standard. *Escherichia coli* NBRC3972 and *Staphylococcus aureus* NBRC12732 were used as test bacteria.

**Fig. 7:**
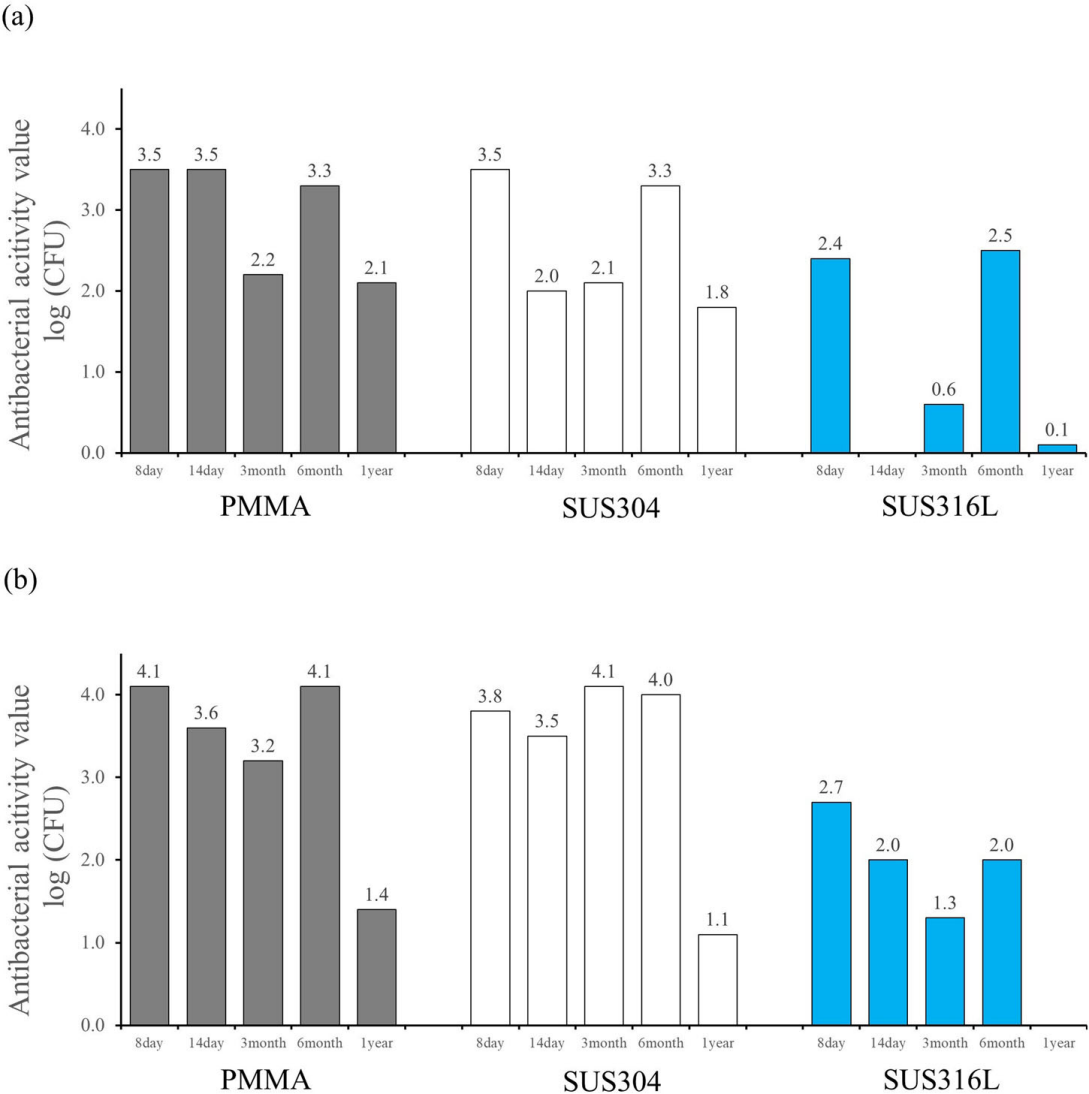
Antimicrobial activity values from 8 days to 1 year after CBMS treatment in PMMA, SUS304 and SUS316L substrates. substrates. **a** *Escherichia coli*, **b** *Staphylococcus aureus*.

Figure 7 shows the changes in antibacterial activity of the PMMA, SUS304, and SUS316L samples coated with CBMS over time. The antibacterial activity values of all the substrates remained above 2.0 for up to 6 months.

The samples were left attached to indoor walls at normal room temperature and pressure without special environmental controls, simulating ordinary daily use conditions. Antibacterial tests were conducted after predetermined time intervals. Unlike typical antibacterial tests, which involve a 24 h culture period, in this test, each sample was cultured with bacteria for only 2 hours to assess its short-term antibacterial properties. Notably, data for *E. coli* on SUS316L after 14 days were not obtained.

Although there were slight differences depending on the type of bacteria, the antibacterial activity values after 3 months showed a minor decrease. Interestingly, in some cases, the results after 6 months were better than those obtained after 3 months. Additionally, the antibacterial activity of the sample on the SUS316 substrate was slightly lower than that of samples on the other substrates.

SUS304 is composed of 18% Cr and 8% Ni, whereas SUS316L contains 18% Cr, 12% Ni, and 2–3% molybdenum (Mo) to enhance corrosion resistance^38^. It remains uncertain whether the difference in antibacterial properties is attributable to the substrate composition.

Overall, the antibacterial activity values of all the substrates remained above 2.0 for up to 6 months, even after only 2 hours of culture with bacteria.

### MRSA sterilization performance and sustainability in a simulated hospital environment

**Fig. 8:**
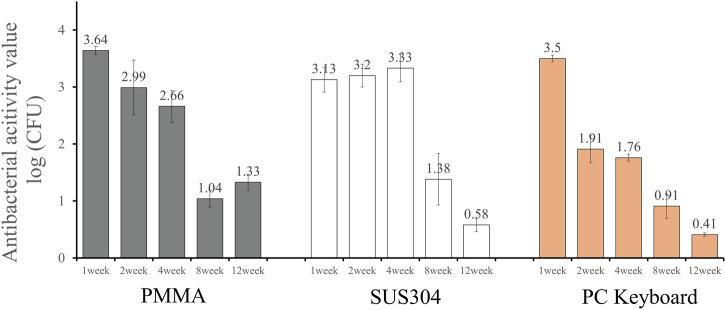
Antibacterial activity values of MRSA on various substrates depending on the time after CBMS treatment.

Figure 8 depicts the changes in antibacterial activity values over time on SUS304, PMMA, and PC keyboards.

At an incubation time of 18 h, the results for MRSA were below the limit of detection, as shown in Table 2. However, the incubation time for this test was set at 2 h because hospitals with many patient visits require rapid antimicrobial performance.

For SUS304 samples, the antibacterial activity value was 3 or higher after 4 weeks of treatment, confirming the sterilization effect; a value of 3 or more was observed even with an incubation time of 2 h. However, after 8 weeks, the antibacterial activity value decreased, and after 12 weeks, it dropped to 0.6.

On the other hand, for PMMA, the antibacterial activity value was 3 or more at 1 week after treatment and remained at 2 or higher up to 4 weeks, but it decreased thereafter. For the PC keyboard, the antibacterial activity value was 3 or higher one week after treatment but consistently remained below 3 after 2 weeks.

Regarding MRSA, although the duration differs from the results shown in Fig. 7, the findings suggest that the persistence of antibacterial activity varies depending on the substrate. The CBMS-coated substrates exhibited good antibacterial activity persistence even when SUS304 was used.

### Product toxicity and safety

Table 4 presents the safety evaluation results of CBMS. The results of the acute oral test (OECD TG423), acute skin irritation test (OECD TG404), skin sensitization test (OECD TG406), and reverse mutation test (GUIDANCE ON GENOTOXICITY TESTING AND DATA INTERPRETATION FOR PHARMACEUTICALS INTENDED FOR HUMAN USE, Pharmaceutical Food and Drug Administration Issue 0920 No. 2, September 20, 2012, Japan) demonstrate that CBMS is highly safe and complies with the safety standards set by SIAA in Japan.

**Table 4:** Biotoxicity properties of CBMS

Test Item		Result	GHS Classification
Acute oral toxicity test	TG423	LD_50_≧2000 mg/kg	Category 5 or Not Classified
Acute Dermal Irritation/Corrosion	TG404	P.I.I. = 0	Not Classified
Skin Sensitization	TG406	Not observed	Not Classified
Reverse mutation test (Ames Test)	–	Negative	Not Classified

### Trial at a sushi restaurant

**Fig. 9:**
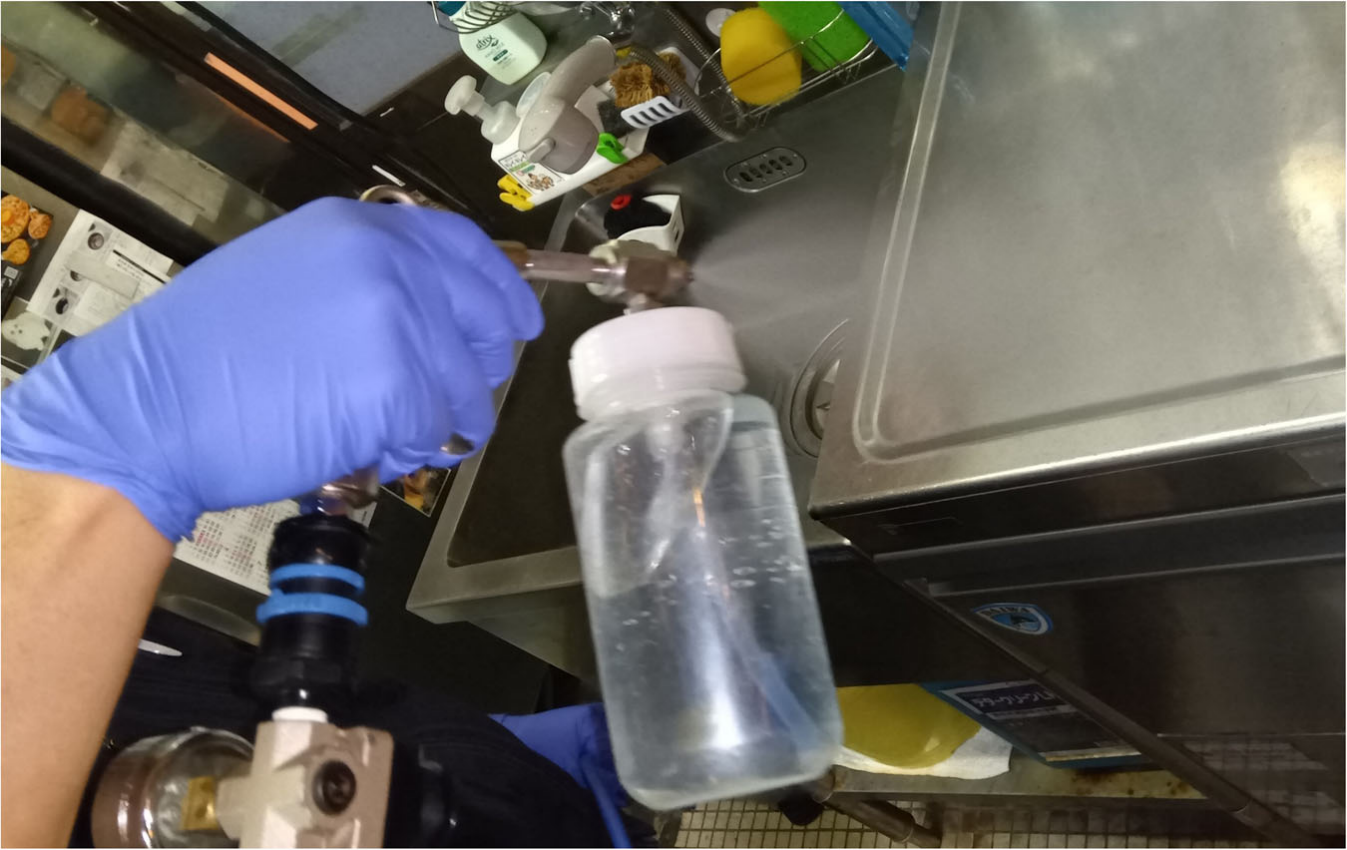
Photo of CBMS being sprayed in a kitchen.

Figure 9 displays a photo of CBMS being sprayed in the kitchen of a sushi restaurant. CBMS was applied at a rate of 20 ml/m2 across various areas of the restaurant. Due to its safe performance, it was sprayed not only in the kitchen area but also on cooking utensils, tables, counters, toilets, and other surfaces. Following application, a waiting period of 48 hours was necessary for drying and solidification.

**Fig. 10:**
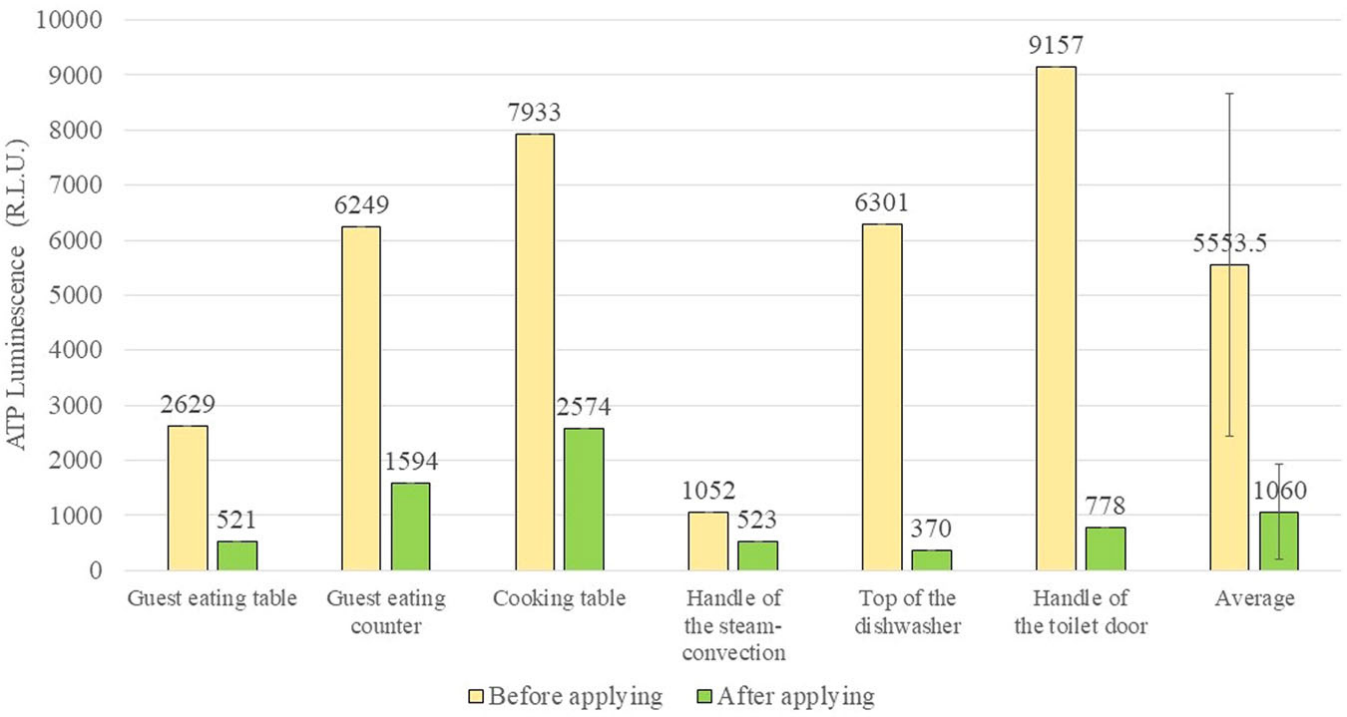
Average ATP (adenosine triphosphate) measurement data at each location in the sushi restaurant before and after CBMS application and drying.

Figure 10 shows the ATP measurement results for each area of the sushi restaurant. Prior to spraying, the average ATP value was approximately 5500, and it subsequently decreased to approximately 1000 after spray drying. This reduction in ATP value suggests an antimicrobial effect of the liquid agent, indicating its potential sterilization capabilities.

## Discussion

Copper has been recognized for its antibacterial properties for many years. Recent research has revealed that copper ions within nanoparticles have the ability to infiltrate microbial cells. This infiltration occurs through the attachment of nanoparticles to bacterial cells, leading to the generation of reactive oxygen species (ROS)^21^. This process is believed to induce elevated levels of intracellular oxidative stress within bacterial cells^39,40^.

Copper ions are naturally present in various foods, and in small amounts, copper serves as an essential trace element necessary for several physiological functions^41,42^. However, excessive intake of copper has been highlighted as potentially harmful to health^43–46^.

The precipitates of CBMS applied at a rate of 20 ml/m^2^ contained various inorganic substances, with the following concentrations: Cu^2+^ : 2.8 mg/l, Ca^2+^ : 0.022 mg/l, Cl^−^ : 0.66 mg/l, HPO_4_^2−^ : 0.24 mg/l. The amount of copper applied in 1 m^2^ is roughly equivalent to the amount found in approximately 450–600 g of liver from food sources. Consequently, the quantity of copper present in the SBSCD, which constitutes the precipitates of CBMS applied in 1 m^2^, is deemed to have negligible effects on the human body^47^.

Immediately after CBMS is applied to a substrate, it is in a liquid state and can be easily wiped off from the substrate. After CBMS dries at room temperature for approximately 24 h, a precipitate with an SBSCD structure forms. Therefore, we believe that the exhibition of antimicrobial properties immediately after application (in the liquid state) is not within the original scope of application of CBMS. However, it is possible to add ethanol to CBMS; it is believed that in this case, the antibacterial and antiviral properties will be immediately effective within a few seconds to a few minutes, depending on the amount of ethanol. This speculation needs to be experimentally confirmed.

SEM observations indicated that CBMS formed an SBSCD structure, with folds ranging in size from approximately 50 to 100 nm. Additionally, TEM and EDS observations of the Zr substrate suggested that copper permeated the substrate and formed strong bonds with it. XPS analysis confirmed the presence of copper in the substrates even after a year of indoor use at room temperature and humidity, with copper oxidation progressing to form CuO, which has antibacterial properties.

Viruses are 20 to 300 nm in size, and since the antiviral effect was exerted in a short period of less than 30 min, it is possible that viruses were adsorbed into the folds of the SBSCD structure mentioned above and lost their activity. On the other hand, typical bacteria are 1 to 10 micrometres in size, which is much larger than the folds of SBSCD, so it is unlikely that bacteria would adsorb to SBSCD. In this study, we did not conduct short-term antibacterial activity tests for the same period as for viruses; considering the size of bacteria and the folds of SBSCD, it is unclear whether an antibacterial effect appears as quickly as it does against viruses.

In this study, no antibacterial tests were conducted with a 24 hour incubation period to assess the immediate antibacterial effect of the CBMS-coated substrate samples. Typically, the longer the bacteria are allowed to incubate on an antimicrobial product, the higher the antimicrobial activity tends to be^48,49^. Therefore, it is suggested that selecting a 24-hour incubation period for antimicrobial testing might lead to improved antibacterial performance.

Since CuO persisted on the substrate even after a year of being left undisturbed, and considering its known antibacterial properties, the treatment is presumed to retain its antibacterial efficacy over an extended duration. Furthermore, the antibacterial component Cu changed over time following application, yet the antibacterial performance remained consistent. There are several potential reasons why the antibacterial effectiveness of CuO may decline over time from the initial level.

- It can be speculated that various compounds containing monovalent copper ions such as Cu_2_O and CuCl, as well as divalent copper ions such as Cu_3_(PO_4_)_2_, Cu_2_P_2_O_7_, CuO, and CuCl_2,_ transformed into CuO, which predominantly consists of divalent copper ions. Since the antibacterial efficacy of divalent copper ions is generally weaker than that of monovalent copper ions, the antibacterial performance may deteriorate over time^48^.
- Copper phosphate compounds such as Cu_3_(PO_4_)_2_ and Cu_2_P_2_O_7_ exhibit photocatalytic activity. This implies that the immediate enhancement of antibacterial properties after application may stem not only from the effect of copper ions but also from the photocatalytic action of copper compounds. However, it is possible that the photocatalytic performance diminishes over time^27,28^.
- XPS analysis revealed alterations in the shape of the precipitates due to oxidation reactions resulting from exposure, leading to a decreased specific surface area caused by secondary aggregation. This phenomenon is believed to contribute to the reduction in antibacterial properties^50,51^.
- Considering points [1], [2], and [3], it is evident that various factors contribute to the potential decline in antibacterial efficacy over time.

From the XPS analysis, crystallization is expected to continue up to a drying time of 6 months, after which the active antibacterial component will be consumed. Therefore, it is expected that the antibacterial effect will show an increasing trend up to 6 months and then a decreasing trend.

Additionally, there is a theory suggesting that doping CuO with transition elements can enhance its antibacterial properties^52^, which opens the possibility for further improvement.

In addition, the antibacterial properties of the samples applied to different substrates varied.

The TEM/EDS results for the sample coated on the Zr substrate revealed strong bonding between the CBMS precipitate (antibacterial component) and the Zr metal, indicating a chemical reaction with the substrate. This suggests that CBMS forms bonds extending from the metal surface into the interior through chemical reactions.

Additionally, a distinct SBSCD structure was evident in the precipitates on acrylic substrates. Thus, even though the antibacterial agent remained the same, the structure of the precipitate appeared to differ slightly depending on the substrate.

On the other hand, even with longer drying times, no differences in the composition of the precipitates were observed between the different substrate types. Therefore, the dependence of antibacterial properties on substrate type is thought to be related to the number and crystallinity of crystallites and not to the type of precipitate. Cu ions are involved in antibacterial properties, and the elution behaviour of Cu ions is thought to differ depending on crystallinity.

Recently, it has come to light that living organisms exhibit diverse functions attributed to variances in their surface nanostructures^53^. Additionally, the literature indicates that antibacterial properties vary depending on these nanostructures. For instance, such differences may arise from physical interactions or the relative contribution of charges^54,55^.

Considering these factors, the variability in antibacterial persistence depending on the substrate material can be attributed to the following factors:

- Whether the precipitate is chemically bonded to the substrate.
- The nanoparticulate structure of the precipitates formed, which varies depending on the substrate. Consequently, the adsorption capacity of bacteria and viruses varies depending on the support material, affecting the antibacterial and antiviral efficacy.
- Differences in antibacterial properties due to variations in the nanostructure of the support substrate itself.

Notably, the samples coated on SUS304, which is commonly used in commercial kitchen countertops, exhibited superior antimicrobial activity both in the comparison of antimicrobial effects across different substrates in section 3 and in the hospital environment assessment in section 4. However, these observations warrant further study and consideration.

There were also variations in antibacterial properties depending on the bacterial species. Differences among bacterial species can stem from variations in their inherent resistance, as well as disparities in culture solutions and protocols. However, such differences were not explicitly tested in this experiment.

Among the antibacterial activity results reported in section 4 for MRSA, which were less effective than expected based on the results in section 3 of this study, it is noteworthy that the antibacterial activity value remained above 3 even after one month of application to the SUS304 substrate. On the other hand, neither the 70% ethanol-based antibacterial agent nor the D-125-based antibacterial agent showed antibacterial effects, as defined by a reduction in bacterial count by 3 log CFU or more, compared with CBMS after more than one week (data not shown). These findings suggest that the antibacterial performance can be sustained by applying the coating once a month.

Toxicological safety testing of a product is typically subject to a minimum requirement. Further testing is necessary for application to assess toxicity to humans or animals. However, on the basis of the typical application amount, the deposited quantity is significantly less than what would affect the human body. Hence, it is suggested that this new antibacterial and antiviral agent is highly safe for health.

The trial conducted at a sushi restaurant was performed with the cooperation of an establishment that has been in operation for over 100 years since the Meiji era.

The ATP method, known for its simplicity and speed, is frequently employed in food manufacturing and medical environments for antibacterial testing^56,57^. This method utilizes an enzyme-cycling technique involving the luminescence reaction of firefly luciferase and pyruvate orthophosphate dikinase (PPDK). By measuring luminescence, which is proportional to the total amount of ATP and AMP, microorganisms and ATP/AMP in the sample can be quantified^58–60^.

Importantly, while this test differs from the culture method and can be executed quickly, it also counts food residue and dead bacteria. The reduction in ATP after CBMS application may be attributed to ATP conversion to other compounds or degradation by CBMS, although the precise mechanism remains unclear.

Nevertheless, since ATP reagents emit light upon reaction with ATP and since ATP is removed by the swab and detected within the system, it is improbable that the rigid SBSCD, which is tightly bound to the substrate, would deactivate the ATP reagents. Regardless, ATP levels decreased after application in real-world environments, suggesting an enhancement in food safety.

The SBSCD precipitate of CBMS maintained its antibacterial activity over an extended period due to its gradual evolution in structure from the initial form, demonstrating effective antibacterial properties even after short culture times.

CBMS with controlled acidity demonstrates high safety for the human body and can be applied to stainless steel materials used in kitchens and tables, particularly in kitchen equipment such as knives, pans, ovens, mixers, etc. Due to its enduring antibacterial effect, continuous application can sustain its antibacterial properties over time.

We anticipate that this SBSCD will contribute to mitigating infection risks for both customers and employees in fast-paced food service settings such as restaurants.

## Methods

### Analysis of the structure and composition of the precipitates

The copper ion mixed solution (CBMS) used in this study is a copper chloride solution prepared by adjusting the pH with phosphoric acid or other substances to precipitate substances containing monovalent copper ions, which form a strong bond with the substrate after drying. Liquid agent-coated substrates was prepared by spraying CBMS at 20 ml/m^2^ onto various substrates (PMMA, SUS304, SUS316L, and Zr), which were left to dry for various periods ranging from 8 days to 1 year at room temperature. Due to the small amount of precipitated product on the substrates after room-temperature exposure, X-ray diffraction could not be performed. Instead, the chemical composition of the outermost layer of the precipitated product was analysed via X-ray photoelectron spectroscopy (XPS) with a microprobe (ULVAC PHI Inc., Kanagawa, Japan). Al Kα radiation served as the X-ray source, and all binding energies were referenced to the carbon 1 s component (284.6 eV). The XPS takeoff angle was set at 45°, allowing the system to detect photoelectrons to a depth of 4–5 nm from the surface. The analysis area was 0.8 mm in diameter.

The surfaces of the CBMS-coated substrates were examined by scanning electron microscopy (SEM) (SU-8000, Hitachi, Co. Ltd., Tokyo, Japan) to analyse the morphology and size of the precipitates. Energy dispersive X-ray spectroscopy (EDX) was used to investigate the chemical composition of the precipitates.

For cross-sectional observation, the CBMS-coated substrates were embedded in resin and thinned using a Hitachi NB5000 focused ion beam SEM system (Hitachi Co., Ltd., Tokyo, Japan) via a microsampling technique to prepare transmission electron microscope (TEM) samples. The substrates coated with CBMS were examined with a JEM-F200 instrument (JEOL Co., Ltd., Tokyo, Japan) at an accelerating voltage of 200 kV, and electron diffraction patterns of the precipitates were analysed.”

### Various antibacterial and antiviral properties

The following tests were conducted to assess the antibacterial and antiviral properties of the CBMS-coated substrates. For antibacterial property assessment, we employed an antibacterial test method for textile products, considering potential applications such as restaurant seats. Antiviral tests were conducted by measuring the antiviral activity on plastics and other nonporous surfaces.

The antibacterial test procedure was as follows: A treated cloth was prepared by spraying 1 ml of CBMS onto a 5 cm × 5 cm standard cotton cloth and drying it in a safety cabinet for 24 h. The cloth was then evaluated using the bacterial liquid absorption method according to ISO 20743:2013. This involved diluting each precultured bacterial solution 20 times with a liquid medium such as nutrient medium (NB) or trypto-soy medium (TSB) to achieve a concentration of 1 × 10^5^ to 3 × 10^5^ CFU/mL. Subsequently, 0.2 mL of the diluted solution was applied to treated and untreated wipes that had been immersed in 99% pure ethanol for 5 min, thoroughly dried, and sterilized. After the addition of 20 mL of SCDLP medium, inactivation solution, or wash-out physiological saline to the cloth immediately after application, the cloth was incubated at 37 ± 2 °C for 18 h. The bacteria were then washed off of the cloth using a tube stirrer. A 10-fold dilution series was prepared, and 1 mL of each dilution was mixed with 15 mL of agar warmed to 45–46 °C, poured into a Petri dish, and cultured at 37 °C ± 2 °C for 48 h, after which the number of colonies (CFU/mL) was measured. The bacteria used included *Escherichia coli* NBRC3301, *Escherichia coli* O157 RIMD 0509952, *Staphylococcus aureus* NBRC12732, methicillin-resistant *Staphylococcus aureus* IID1677, *Pseudomonas aeruginosa* NBRC3080, *Moraxella osloensis* ATCC19976, *Klebsiella pneumoniae* NBRC13277, *Salmonella enterica* subsp. Enterica NBRC3313, and *Micrococcus luteus* NBRC3333.

The antiviral assay procedure was as follows: A treated polyethylene film sample was prepared by spraying 1 ml of CBMS onto a 5 cm × 5 cm polyethylene film and drying it in a safety cabinet for 24 h. The test samples were evaluated using the plaque measurement method based on the ISO 21702 test method. This involved applying 0.4 mL of virus suspension onto a polyethylene film sample and placing the sample in close contact with another sterilized 4 cm × 4 cm polyethylene film to prevent the suspension from drying. The samples were allowed to react at 25 °C for 15, 30, or 60 minutes. After the reaction, the film was recovered from the sample, and 10 mL of washout solution was added to remove the virus from the sample and recover the film. The collected viruses were used to infect host cells, and after culturing for 2 to 3 days, the virus infectivity titre was measured. The viruses used included influenza A H3N2 (A/Hong Kong/8/68; TC adapted ATCC VR-1679), Feline calicivirus (FCV) (strain F-9 ATCC VR-782), SARS-CoV-2 (NIID isolate JPN/TY/WK-521), the SARS-CoV-2 alpha mutant strain (hCoV-19/Japan/QK002/2020), the SARS-CoV-2 delta mutant strain (hCoV-19/Japan/TY11-927-P1/2021), and the SARS-CoV-2 omicron mutant strain (hCoV-19/Japan/TY38-873/2021). Influenza A and FCV were allowed to react for 15 minutes, the omicron strain of SARS-CoV-2 was allowed to react for 60 minutes, and the other viruses were allowed to react for 30 minutes.

### Sustainability of the antibacterial effect of the liquid agent against *Escherichia coli* and *Staphylococcus aureus* on PMMA, SUS304, and SUS316L

Samples were evaluated using the film adhesion method in accordance with JIS Z2801 (ISO 22196:2007).

PMMA, SUS304, and SUS316L were selected as the base materials, each measuring 50 mm × 50 mm × 1 mm. CBMS was applied at a rate of 20 ml/m^2^ to each substrate. Following application, the samples were left at room temperature and humidity for 8 days, 14 days, 3 months, 6 months, or 1 year.

Subsequently, 0.4 ml of 1/500 NB bacterial solution was applied to the samples, which had been immersed in 99% pure ethanol for 5 minutes, thoroughly dried, and sterilized in accordance with JIS Z 2801. Each sample was then placed in close contact with a sterilized 4 cm × 4 cm polyethylene film and cultured for 2 hours at 35 ± 1 °C and a relative humidity of 90% or higher. No contamination from other bacteria was detected at the end of the test.

After incubation, the test bacteria were washed off of the materials with 10 ml of SCDLP medium. A 10-fold dilution series was prepared, and then the bacteria were cultured on standard agar medium for 40 to 48 h at 35 ± 1 °C. The bacteria used in the test included *Escherichia coli* NBRC3972 and *Staphylococcus aureus* NBRC12732]

### MRSA sterilization performance and sustainability in a simulated hospital environment

SUS304, PMMA, and a PC keyboard were chosen as the base materials, each measuring 20 mm × 20 mm × 0.1 mm. UV sterilization treatment was applied for 30 minutes on one side and 30 minutes on the other side of each material.

Subsequently, CBMS was sprayed at a rate of 20 ml/m^2^ and dried at room temperature, and untreated samples were also prepared; the samples were allowed to stand for 1 week, 2 weeks, 4 weeks, 8 weeks, or 12 weeks.

One hundred microlitres of MRSA ATCC 43300 bacterial solution at a concentration of 10^8 CFU/mL was applied to the entire surface of each material in a Petri dish. The Petri dishes were covered and stored at 22 ± 2 °C and 30–40% RH for 2 h. Following this, each material was suspended in 5 mL of sterile saline. The test bacteria were washed away from the materials; then, a 10-fold dilution series was prepared, and 100 μL of each dilution was plated onto mannitol salt agar (SHIMADZU, Kyoto, Japan). The agar plates were cultured at 35 °C for 18 h, after which the number of colonies (CFU) was counted.

Each base material was analysed with *N* = 5 replicates, and the average value (log CFU) and standard deviation (SD) were calculated using the data from *N* = 3 replicates, excluding the minimum and maximum data points.

The antibacterial activity value was calculated using the following formula:

$${\rm{Antibacterial\; activity\; value}}=(\log {\rm{value\; of\; average\; CFU\; of\; untreated\; sample}})-(\log {\rm{value\; of\; average\; CFU\; of\; CBMS}}-{\rm{treated\; sample}})$$

The presence or absence of a bactericidal effect was determined according to the guidelines of the U.S. Environmental Protection Agency (EPA), with an antibacterial activity value of 3 or higher indicating a bactericidal effect.

### Trial at a sushi restaurant

CBMS was sprayed at a rate of 20 ml/m^2^ across various areas of the sushi restaurant. It was applied not only to the kitchen area but also to cooking utensils, tables, counters, toilets, and other surfaces.

For testing, we employed a Lumitester^TM^ with a Lucipac^TM^ Pen as the test reagent (Kikkoman Biochemifa Company, Tokyo, Japan). Initially, a 10 cm × 10 cm area in each designated zone was uniformly wiped with a Lucipac^TM^ Pen.

Measurements were then taken at the same locations both before the application of the liquid agent and 8 h after application.

## Data Availability

The authors declare that the data supporting the findings of this study are available within the articles.
